# Trends and Behavioral Correlates of Excessive Screen Time Among Swedish Adolescents: A Repeated Cross-Sectional Study (2017–2023)

**DOI:** 10.1016/j.abrep.2026.100672

**Published:** 2026-01-29

**Authors:** Amir Pakpour, Karina Huus, Daniel Kwasi Ahorsu, Gunilla Björling, Anders Broström, Staffan Bengtsson, Malin Jakobsson, Marit Eriksson

**Affiliations:** a Department of Nursing, School of Health and Welfare, Jönköping University, Jönköping, Sweden; b Futurum- the Academy for Health and Care, Region Jönköping County, Jönköping, Sweden; c Department of Health, Medicine and Caring Sciences, Linköping University, Linköping, Sweden; d Department of Special Education and Counselling, The Education University of Hong Kong, Hong Kong, China; e Department of Neurobiology Care Sciences and Society, Karolinska Institutet, Stockholm, Sweden; f School of Nursing, KCMC University, Moshi, Tanzania; g Department of Clinical Neurophysiology, Linköping University Hospital, Linköping, Sweden; h Department of Health and Caring Sciences, Western Norway University of Applied Sciences, Bergen, Vestlandet, Norway; i CHILD-research group, Department of Nursing Science, School of Health and Welfare, Jönköping University, Jönköping, Sweden; j Department of Social Work, School of Health and Welfare, Jönköping University, Jönköping, Sweden

**Keywords:** Swedish adolescents, Screen time, Risky behaviors, Sleep duration, Physical activity, Cross-sectional survey, family structure

## Abstract

**Aim:**

This repeated cross-sectional study examined trends and behavioral correlates of excessive screen time among Swedish adolescents across 2017, 2020 and 2023. Specifically, this study examined 1) temporal trends of variables used, 2) correlates of pooled screen time, and 3) correlates of specific screen modalities (gaming, social media, and film/TV viewing).

**Methods:**

Data were collected from n=8,300 upper secondary school students in Jönköping County (n=2,319 in 2017; n=3,056 in 2020; n=2,925 in 2023). Measures used included risk behaviors (e.g., alcohol, smoking), physical activity, psychosocial trust, exposure to violence and victimization, sleep duration, psychosomatic symptoms, absenteeism and living arrangement. General linear models (GLM; UNIANOVA) were conducted, adjusting for relevant covariates.

**Results:**

Alcohol use, smoking, shorter sleep duration, higher psychosomatic symptoms, and school absenteeism were associated with higher pooled screen time, whereas physical activity, female gender, and living with both parents were associated with lower pooled screen time. Across modality-specific analyses, shorter sleep duration, lower physical activity, living without both parents, and school absenteeism were consistently associated with higher screen use across gaming, social media, and film/TV viewing, while substance-use and gender associations varied by modality.

**Conclusion:**

Sleep, physical activity, family structure, and school absenteeism appear to be robust correlates across screen modalities, supporting the importance of targeting these factors in adolescent health promotion and digital media guidance. These findings highlight the need for parents, researchers, and health professionals to pay closer attention to adolescents’ digital media use.

## Highlights

• High levels of weekday after-school screen time remained common across all survey waves.
• Shorter sleep duration and substance use, particularly alcohol use, were associated with higher overall screen time across survey waves.
• Higher physical activity and longer sleep duration were consistently associated with lower use across screen modalities.
• Risk behaviors were most strongly associated with social media use, with weaker and more heterogeneous associations observed for gaming and film/TV viewing.
• Screen-use correlates differed by modality, underscoring the importance of distinguishing between gaming, social media, and film/TV use in adolescent research.

## 1 Introduction

The emergence and advancement of technological devices have improved various aspects of our lives, making them integral to our day-to-day activities (Caballero-Julia et al., 2024). Children and adolescents are not exempted from this digital-saturated world, which makes them dependent on these digital devices for activities such as social media use and gaming. Screen time is used to describe the time that an individual spends on screen-based entertainment such as television, video games, video clips, social media, and streamed films but excludes school-related screen use programs or apps serving as aids for individuals with functional difficulties ([AuthorError] et al., 2013, Oswald et al., 2020; Public Health Agency of Sweden, 2024). For adolescents, screen time of more than two to three hours per day is deemed problematic (Council on Communications and Media et al., 2013; Public Health Agency of Sweden, 2024; Ramirez et al., 2011). Screen time, as used here, is not a proxy for overall sedentary behavior (read Zhang et al., 2025 for more details) but just time spent on screen.

The impact of excessive screen time among adolescents is evident in its associations with various physical (Kharel et al., 2022, Lua et al., 2023, Nyberg et al., 2023, Priftis and Panagiotakos, 2023, Zou et al., 2022), social (Gao and Gao, 2024, Primack et al., 2017, Sanders et al., 2024), and mental health problems (Kharel et al., 2022, Lua et al., 2023, Nagata et al., 2024, Sanders et al., 2024, Santos et al., 2023, Tang et al., 2021). The displacement hypothesis suggests that time spent on one activity (e.g., watching screens) reduces/displaces the time available for other activities (e.g., physical activity and sleep), leading to potential negative consequences (Lee, 2009, Lo et al., 2025, Neuman, 1991, Roberts et al., 1993). Subsequently, excessive screen time lowers an individual’s self-regulation skills, which are the effortful control over one’s thoughts, emotions, choices, impulses, and behaviors (Howard et al., 2025).

Several studies, including among Swedish adolescents, have examined the factors associated with or predicting screen time among adolescents (Frielingsdorf et al., 2025, Hökby et al., 2025, Kjellenberg et al., 2022, Shalani et al., 2021, Song and Kim, 2022). Some factors, namely having a non-intact family, poor academic performance, loneliness and drinking carbonated beverages were positively associated with high screen time, while having higher parental education and being physically active were negatively associated with high screen time (Krist et al., 2020, Shalani et al., 2021, Wang et al., 2018). Other correlates of high screen time included being male, having a mother with higher education, and having a history of school failure as an adolescent (Dumith et al., 2012, Krist et al., 2020, Shalani et al., 2021). Also, there were gender differences in overall screen time and the type of digital media use (Busschaert et al., 2015, Dumith et al., 2012, Shalani et al., 2021). Moreover, there may be several unexplored factors that predict excessive screen time, which need to be examined.

Different studies have examined distinct factors associated with or predicting excessive screen time among children and/or adolescents. These factors can be broadly categorized into 1) child biological and demographic correlates, 2) behavioral correlates, 3) family biological and demographic correlates, 4) family structure-related correlates, and 5) socio-cultural and environmental correlates (Shalani et al., 2021). However, there are inconsistencies in some findings, for example concerning gender, digital device, and purpose of screen use. Also, there are limited reports on adolescents’ risk behavior influencing excessive screen time. Furthermore, a look into the correlates of screen time using a large sample size over multiple years (including the post-COVID-19 era) would help identify long-term, stable correlates of excessive screen time. Therefore, the present study extends prior research based on the displacement hypothesis (Lee, 2009, Lo et al., 2025, Neuman, 1991, Roberts et al., 1993) and self-regulation theory (Howard et al., 2025, Liau et al., 2015). Using these theories and three large Swedish adolescent datasets from 2017, 2020, and 2023, the present study examines the 1) temporal trends in screen time, health and risky behaviors across three time waves, 2) correlates of pooled screen time (>3 hours per weekday after school), and 3) correlates of modality-specific screen use*.*

## 2 Method

### 2.1 Participants and procedures

Data for the present study were collected through three cross-sectional surveys conducted in 2017, 2020, and 2023 in Jönköping County, Sweden. These data are part of the Youth Public Health Survey organized by the Region Jönköping County.

This project received a non-binding advisory opinion from the Swedish Ethical Review Authority, which assessed that the data were collected anonymously and do not constitute personal data under the Swedish Ethical Review Act (2003:460). Accordingly, the project did not require formal ethical review and was assessed as having no obstacles to the planned research (Dnr 2020-03173). The present study is based on secondary analyses of data from the Youth Public Health Survey conducted by Region Jönköping County. All data were fully anonymized by the data owner prior to being made available to the research team, and no personal identifiers were accessible at any stage of the analysis. As a result, individual participants could not be identified, and no reasonably likely re-identification was possible. Data access was restricted and governed by the data owner, and only aggregated results are reported. All participants were upper secondary school students, primarily aged 16–17 years. Inclusion criteria required participants to be at least 15 years old, in accordance with §18 of the Swedish Ethical Review Act (2003:460), which allows adolescents aged 15 years and older to provide independent informed consent for survey-based research. Participation in the original survey was voluntary, and all participants provided informed online consent prior to completing the anonymous questionnaire, with the right to withdraw at any time without providing a reason.

The questionnaire was distributed in a school setting, where students received a link along with information about the study's aims. Those who agreed to participate completed the survey anonymously during school hours. The response rate for each year for 2nd-year upper secondary students was 68% (2338/3452) for 2017, 77% (3182/4251) for 2020, and 77% (3108/4058) for 2023.

Portions of the data have been used in earlier publications addressing different research questions and subsamples; these studies are cited for transparency (Pakpour et al., 2024; Pakpour et al., 2025a, Pakpour et al., 2025b).

### 2.2 Study measures

The data collection was carried out using a self-reported questionnaire containing several sections:

#### 2.2.1 Demographic Information

Gender and living arrangements (e.g., whether they lived with both parents).

#### 2.2.2 Risk Behaviors

These were measured with single-item questions on snus (moist tobacco) use, smoking, alcohol consumption in the past 12 months, and drug use (e.g., “Have you ever used narcotics?”).

#### 2.2.3 Physical Activity

This was assessed using one item asking how often students engaged in at least 30 minutes of exercise during leisure time that made them sweat and become short of breath. Response options included: “Every day”, “4–6 times per week”, “2–3 times per week”, “Once per week”, “1–3 times per month”, “Less than once per month”, and “Never”. Adolescents who reported “2–3 times per week” or more were classified as physically active.

#### 2.2.4 Psychosocial trust

Psychosocial trust was assessed using two items: whether students believed they could always rely on at least one parent or guardian, and whether they had trustworthy adults in their environment. Items were rated on a five-point Likert scale ranging from 1 (strongly disagree) to 5 (strongly agree), and a total score was computed. Internal consistency for this two-item measure was acceptable across all years (Cronbach’s α = 0.77 in 2017, 0.65 in 2020, and 0.713 in 2023).

#### 2.2.5 Violence exposure and victimization

Students were asked whether they had ever experienced physical or psychological violence from an adult or had been victims of bullying or harassment. Each was assessed using a single-item question.

#### 2.2.6 Sleep duration

Measured with a single item asking students how many hours they usually sleep per night during the week.

#### 2.2.7 Psychosomatic symptoms

These were captured through eight items assessing common complaints such as headaches, stomach pain, back pain, sadness, irritability, anxiety, stress, and difficulty falling asleep. Items were rated on a five-point scale from 1 (rarely or never) to 5 (almost every day), and scores were summed to yield a total symptom score. Internal consistency was excellent (Cronbach’s α = 0.866 in 2017, 0.864 in 2020, and 0.861 in 2023).

#### 2.2.8 School absenteeism

School absenteeism was measured using a single item on skipping school

#### 2.2.9 Screen time

Screen time was assessed using three questions that asked students to estimate how many hours per day, on weekdays after school, they typically spent on (1) playing video games on a TV, computer, or mobile devices, (2) watching films, series, TV programs, or video clips (e.g., on YouTube), and (3) using social media. Each item was rated on a five-point Likert scale, with the following response options: 1 = less than one hour, 2 = Between one and two hours, 3 = Between two and four hours, 4 = Between five and six hours, and 5 = Seven hours or more. To estimate total screen time the categorical responses were converted to weighted midpoint values. Specifically, students who reported less than one hour spent on an activity were assigned a value of 0.5 hours; those reporting one to two hours were assigned 1.5 hours; reports of two to four hours were assigned three hours; five to six hours corresponded to 5.5 hours; and those indicating seven or more hours were assigned seven hours. These values were then summed across all three activity types to produce a composite screen time score for each participant. The internal consistency of this three-item index was modest but acceptable, with Cronbach’s alpha coefficients of 0.620 in 2017, 0.552 in 2020, and 0.614 in 2023. In the present study, excessive screen time was operationalized using two alternative thresholds: ≥3 hours/day and ≥5 hours/day of weekday after-school screen time.

### 2.3 Statistical analysis

All analyses were conducted in IBM SPSS Statistics (version 31) and R version 4.5.2. Screen use outcomes were treated as continuous variables representing weekday after-school leisure screen time (hours), derived from category midpoints and aggregated across gaming, social media, and film/TV viewing. Descriptive statistics were calculated for each survey wave.

To evaluate temporal differences and correlates of screen time, we used pooled general linear models (GLM; UNIANOVA) combining data from 2017, 2020, and 2023, with survey year entered as a categorical factor (reference: 2017). Priori covariates were selected based on conceptual relevance and prior literature and were entered simultaneously to avoid data-driven predictor screening.

To assess whether key associations differed across survey waves, interaction terms between survey year and selected predictors (gender, alcohol use, and sleep duration) were tested. Interaction effects are presented as omnibus tests based on Type III sums of squares. Estimated marginal means by survey year were derived from the fitted models at the mean values of covariates.

Multicollinearity diagnostics indicated no problematic multicollinearity (VIFs within acceptable ranges). Statistical significance was defined as p < .05 (two-sided).

### 2.4 Missing data and sensitivity analyses

Primary analyses were conducted using complete-case estimation (listwise deletion within each model). To assess the robustness of findings to missing data, multiple imputation by chained equations (MICE) was performed as a sensitivity analysis. Twenty imputed datasets were generated using 20 iterations. Continuous variables were imputed using predictive mean matching, and binary variables (alcohol use, smoking, snus use, narcotics use, physical activity, school absenteeism, victimization, exposure to violence, and living with both parents) were imputed using logistic regression. Survey year and gender were treated as design variables and were not imputed.

The imputation model included all variables used in analytic models. Regression estimates from the imputed datasets were pooled using Rubin’s rules. Interaction effects were specified in the analysis models after imputation and estimates for year-specific interaction terms (e.g., 2020 × exposure, 2023 × exposure) were pooled across imputations using Rubin’s rules. Results from the multiple-imputation analyses were compared with complete-case estimates to evaluate consistency in the direction, magnitude, and statistical inference of associations.

#### 2.4.1 Sensitivity analyses using alternative outcome definitions

In addition to the primary analysis treating composite weekday after-school screen time (hours) as a continuous outcome, we conducted sensitivity analyses using binary outcomes to examine robustness to alternative operational definitions of high screen time. High screen time was defined as (i) >3 hours/day and (ii) >5 hours/day on the composite screen-time measure. For each threshold, multivariable logistic regression models were fitted including the same covariate set as in the primary analysis. Adjusted odds ratios (ORs) with 95% confidence intervals are reported. For each binary outcome, both complete-case estimates and pooled estimates from multiple imputation (20 imputations, combined using Rubin’s rules) are presented (Supplementary Table S2).

## 3 Results

### 3.1 Sample Characteristics

In total, n=8,300 upper secondary school students responded to the survey: n=2,319 in 2017, n=3,056 in 2020, and n=2,925 in 2023. Descriptive characteristics of the study participants in the present survey are displayed in Table 1.

**Table 1:** Descriptive Characteristics of Upper Secondary School Students in 2017, 2020, and 2023

**Variable**	**2,017 (n=2,319)Mean ±SD**	**2,020 (n=3,056)Mean ±SD**	**2,023 (n=2,925)Mean ±SD**	**P-value**
Screen time (h/day)	7.63±4.13	7.27±3.64	6.90±3.70	<0.001
Excessive screen time (>3 h/day)	1,997 (86.1%)	2,697 (88.3%)	2,539 (86.8%)	0.031
Sleep duration (h/night)	6.99 ±1.27	7.16±1.20	7.13±1.22	<0.001
Smoking (daily or sometimes)	487 (21.0%)	543 (17.8%)	379 (13.0%)	<0.001
Snus use (daily or sometimes)	297 (12.8%)	723 (23.7%)	716 (24.5%)	<0.001
Ever used narcotics	160 (6.9%)	193 (6.3%)	156 (5.3%)	0.077
Alcohol use (past 12 months)	1,424 (61.4%)	1,836 (60.1%)	1,804 (61.7%)	0.400
Living with both parents	1,599 (69.0%)	2,155 (70.5%)	2,051 (70.1%)	0.627
School absenteeism	777 (33.5%)	860 (28.1%)	918 (31.4%)	<0.001
Psychosocial trust	9.41±1.26	9.20±1.46	9.21±1.44	<0.001
Exposed to violence (physical and/or psychological) by an adult	178 (7.7%)	261 (8.5%)	207 (7.1%)	0.122
Victimization (e.g., bullied or harassed)	393 (16.9%)	247 (8.1%)	274 (9.4%)	<0.001
Gender (boys)	1,128 (48.6%)	1,557 (50.9%)	1,428 (48.8%)	0.177
Regular physical activity	1,546 (66.7%)	1,628 (53.3%)	2,074 (70.9%)	0.001
Psychosomatic symptoms	19.31 ±7.07	19.16 ±7.02	17.37 ±6.33	<0.001
≥2 psychosomatic symptoms	881 (42.5%)	1,168 (41.7%)	1,273 (44.4%)	0.287

### 3.2 Temporal Trends in Screen Time

One-way ANOVA tests revealed statistically significant differences over time in mean screen time, sleep duration, and psychosomatic symptoms (all p <.001). Specifically, the mean weekday screen time decreased from 7.63 hours per weekday after school in 2017 to 6.90 hours per weekday after school in 2023 (p <.001), while psychosomatic symptom scores decreased significantly from 19.31 to 17.37 over the same period. Conversely, average sleep duration increased modestly but significantly, from 6.99 hours/night in 2017 to 7.13 hours/night in 2023 (p <.001). Bonferroni-corrected post hoc tests showed that all pairwise year comparisons were significant for screen time and psychosomatic symptoms, while sleep duration differed significantly between 2017 and both 2020 and 2023, but not between 2020 and 2023.

Chi-square tests showed significant year-to-year changes in several categorical behaviors. The prevalence of excessive screen time (>3 hours per weekday after school) remained high in all years (86.1%–88.3%) but differed slightly across cohorts (p =.031). Smoking (daily or occasional) decreased from 21.0% in 2017 to 13.0% in 2023 (p <.001), while snus use increased sharply, from 12.8% to 24.5% (p <.001). The proportion of students reporting school absenteeism differed significantly (p <.001), as did the proportion of those reporting regular physical activity (p =.001), which varied across survey waves. Victimization showed a significant decrease from 16.9% in 2017 to 8.1% in 2020, followed by a slight increase to 9.4% in 2023 (p <.001).

Gender differences were consistent across all years: girls reported substantially higher rates of ≥2 psychosomatic symptoms than boys (e.g., 2023: 61.5% of girls vs. 26.5% of boys). These patterns remained stable from 2017 to 2023 (data not shown in the table), highlighting persistent gender disparities in subjective health complaints.

### 3.3 Correlates of Screen Time

In pooled general linear models combining the 2017, 2020, and 2023 survey waves, survey year was not independently associated with weekday after-school screen time after adjustment for covariates (2023 vs 2017: B = −1.16, 95% CI −2.63 to 0.32, p = .124; 2020 vs 2017: B = −0.52, 95% CI −2.04 to 1.00, p = .502) (see Table 2). Overall, the model explained about 11% of the variance in screen time (R^2^ = 0.109; adjusted R^2^ = 0.105).

**Table 2:** Pooled general linear model predicting weekday after-school screen time (hours)

**Predictor**	**B**	**SE**	**95% CI**	**p-value**
**Survey year**				
2023 vs 2017	−1.16	0.75	−2.63 to 0.32	.124
2020 vs 2017	−0.52	0.77	−2.04 to 1.00	.502
**Substance use**				
Alcohol use	0.58	0.19	0.22 to 0.94	.002
Smoking	0.76	0.14	0.48 to 1.03	<.001
Snus use	0.21	0.13	−0.04 to 0.45	.095
Narcotics use	0.24	0.21	−0.17 to 0.64	.258
**Sleep & health**				
Sleep duration (hours)	−0.41	0.07	−0.55 to −0.26	<.001
Psychosomatic symptoms	0.04	0.01	0.02 to 0.06	<.001
**Sociodemographic & psychosocial factors**				
Female gender	−0.72	0.18	−1.08 to −0.37	<.001
Living with both parents	−0.49	0.11	−0.69 to −0.28	<.001
School absenteeism	0.57	0.10	0.37 to 0.78	<.001
Physical activity	−1.05	0.10	−1.25 to −0.86	<.001
Psychosocial trust	−0.06	0.04	−0.13 to 0.01	.110
Victimization	0.20	0.19	−0.18 to 0.58	.310
Exposure to violence	0.03	0.22	−0.40 to 0.47	.891
**Interactions (omnibus tests)**				
Year × Gender	—	—	—	.014
Year × Alcohol use	—	—	—	.039
Year × Sleep duration	—	—	—	.476

Notes:

Dependent variable: Weekday after-school screen time (continuous, hours)

Model: Pooled GLM including 2017, 2020, and 2023R^2^ = 0.109 (Adjusted R^2^ = 0.105)

2017 is the reference year. Coefficients represent unstandardized effects (B). Interaction terms are reported as omnibus tests. Sleep duration is measured in hours. Screen time refers to weekday after-school leisure use.

Several behavioral and health-related correlates showed robust associations with higher screen time. Adolescents reporting alcohol use (B = 0.58, 95% CI 0.22 to 0.94, p = .002) and smoking (B = 0.76, 95% CI 0.48 to 1.03, p < .001) had higher screen time, whereas associations for snus use (B = 0.21, 95% CI −0.04 to 0.45, p = .095) and narcotics use (B = 0.24, 95% CI −0.17 to 0.64, p = .258) were not statistically significant. Shorter sleep duration was associated with higher screen time (B = −0.41 per additional hour of sleep, 95% CI −0.55 to −0.26, p < .001), and higher psychosomatic symptom scores were also associated with greater screen time (B = 0.04 per one-unit increase, 95% CI 0.02 to 0.06, p < .001).

Omnibus interaction tests indicated that the associations between screen time and gender (year × gender, p = .014) and alcohol use (year × alcohol use, p = .039) varied across survey waves, whereas there was no evidence that the association between sleep duration and screen time differed by year (year × sleep duration, p = .476).

### 3.4 Sensitivity analysis using multiple imputation

Results from the multiple imputation analyses were largely consistent with those obtained from complete-case models (Supplementary Table S2). The direction and magnitude of associations for key predictors—including substance use behaviors, sleep duration, psychosomatic symptoms, school absenteeism, physical activity, gender, and family structure—remained similar across approaches.

Minor differences in statistical significance were observed for a small number of variables, with some effects reaching conventional significance levels only in the imputed analyses. These differences did not alter the overall pattern of results or the main conclusions of the study.

### 3.5 Sensitivity analyses using binary thresholds.

Sensitivity analyses using binary thresholds (>3 and >5 hours/day) yielded patterns broadly consistent with the primary continuous model. In both complete-case and multiple-imputation analyses, alcohol use, school absenteeism, psychosomatic symptoms, and shorter sleep duration were consistently associated with higher odds of high screen time, whereas physical activity was inversely associated with high screen time (Supplementary Tables S3). Survey year was not associated with high screen time under either threshold. Associations for smoking and living with both parents were more pronounced for the ≥5-hour threshold than for >3 hours/day, suggesting that these factors may be more relevant for very high levels of screen time.

### 3.6 Correlates of Specific Screen Modalities

In pooled general linear models across 2017, 2020, and 2023, mean weekday after-school screen time showed modest decrease for film/TV viewing (2.59 hours in 2017 to 2.31 hours in 2023) and gaming (1.80 hours in 2017 to 1.61 hours in 2023), whereas social media use remained broadly stable (3.18 hours in 2017; 3.00 hours in 2023) (see Supplementary Table S1a-S1c). However, after multivariable adjustment, survey year was not independently associated with any modality-specific outcome.

Across modalities, shorter sleep duration was consistently associated with higher use (film: B = −0.108; gaming: B = −0.135; social media: B = −0.168; all p < .001), and psychosomatic symptoms were positively associated with film/TV viewing and social media use (both p < .001) but not with gaming. Living with both parents was associated with lower use across all three modalities (all p ≤ .011), and higher physical activity was consistently associated with lower use (all p < .001). School absenteeism was positively associated with each modality (all p ≤ .005).

Associations differed by modality for substance-use indicators. For film/TV viewing, narcotics use was positively associated (B = 0.235, p = .008), whereas alcohol use was not. For gaming, alcohol use was inversely associated (B = −0.174, p = .028). For social media, alcohol use, smoking, and snus use were all positively associated (all p < .001). Gender showed strong modality-specific patterns: gender was not associated with film/TV viewing but was strongly associated with gaming (B = −1.357, p < .001), and was positively associated with social media use (B = 0.602, p < .001).

Omnibus interaction tests indicated that associations varied across survey waves for gender in gaming (year × gender, p = .004) and in social media (p = .003), and for alcohol use in social media (year × alcohol use, p = .005), whereas interactions with sleep duration were not statistically significant in any modality-specific model.

## 4 Discussion

The present repeated cross-sectional study examined the trends and correlates of excessive screen time among Swedish adolescents in the years 2017, 2020, and 2023. Trend analyses revealed that there were significant differences in mean screen time, sleep duration, and psychosomatic symptoms over time. Specifically, the mean screen time decreased over time. However, these means are high, reflecting the high prevalence of excessive screen time (>3 hours per weekday after school) in all years (86.1%–88.3%). Moreover, the observed reduction in screen time between 2020 and 2023 may reflect a post-pandemic stabilization of digital behaviors (e.g., in-person schooling and social activities) rather than a sustained reversal of long-term trends. Conversely, average sleep duration increased over time although remained lower than the recommended 8–10 hours’ sleep duration for adolescents (Hirshkowitz et al., 2015). Moreover, psychosomatic symptoms, smoking behavior, and victimization (e.g., bullied or harassed) significantly decreased over time, while snus use and school absenteeism increased over time. Generally, these trends indicate that Swedish adolescents may be aware of good health practices, as observed in increased regular physical activity and sleep duration and decreased screen time over time. Sometimes, the increment in unhealthy habits may be because of maladaptive coping strategies, especially during stressful periods such as the COVID-19 pandemic (Choi et al., 2023, Madigan et al., 2022, Singh et al., 2021, Trott et al., 2022, Delisle Nyström et al., 2023, Kägi-Braun et al., 2025). Also, total screen time should not be equated with total sedentary time as the time may be reallocated to non-screen sedentary activities such as studying, reading, or passive socializing (Zou et al., 2024). It is therefore possible that reductions in screen time were partially offset by increases in non-screen sedentary activities, which were not captured in the present study. Future research integrating comprehensive time-use or accelerometer-based measures would be needed to clarify whether observed changes in screen time translate into meaningful shifts in overall physical activity or sedentary behavior.

The pooled data revealed that alcohol use, smoking, shorter sleep duration, school absenteeism and higher psychosomatic symptoms were associated with higher screen time which indicate that adolescents who engage or have these symptoms may likely use the screen for longer time period which may not be healthy for them. However, being a female adolescent, living with both parents, and having physical activity were associated with lower screen time. It would be worthwhile for researchers to further examine the qualitative reasons underlying why these factors affect screen time. Knowing the reasons behind these predictive factors may help with inhibiting adolescents getting addicted to screens and its associated negative consequences. However, it can be rationalized that factors such as physical activity may likely reduce screen time as it may occupy adolescents from engaging in screen time activities apart from the fact that physical activity serves as adaptive coping strategies. This supports the displacement hypothesis (Buelow et al., 2022, Hopkinson et al., 2024, Lee, 2009, Lo et al., 2025, Neuman, 1991, Roberts et al., 1993). Nonetheless, snus use, narcotics use, psychosocial trust, victimization, and exposure to violence were not associated with screen time in adjusted models which suggest that these factors do not significantly influence screen time in any way among adolescents in Sweden.

Additionally, although survey year (i.e., 2017, 2020, and 2023) was not independently associated with any modality-specific screen use, shorter sleep duration, living with both parents, higher physical activity, and school absenteeism were consistently associated with all the modalities (i.e., film/TV, gaming, and social media). These findings indicate the needed attention to these factors to help deal with extended screen time. This is important as adolescents significantly engage in at least one of these modalities daily and so positively dealing with these factors may help mitigate the extended period of screen time use. It seems quite logical that shorter sleep duration, lower physical activity, and school absenteeism may influence longer screen time but reports on living with both parents are complex. It can be reasoned that living with both parents, especially in stable, married families, may be associated with less overall screen time as two parents offer more investment, supervision, and alternative activities, reducing reliance on screens for engagement. However, personal and media parenting practices (i.e., attitudes towards screen time, screen-time modeling, mealtime screen use, and use of screens to control behavior) may also influence their ward’s screen time (Chong et al., 2023, Ishii et al., 2022, Tang, L., Darlington, G., Ma, D. W. L., Haines, J., & Guelph Family Health Study et al., 2018). Psychosomatic symptoms were associated with film/TV viewing and social media use, whereas gender was associated with gaming and social media use suggesting the need for equal attention as those associated with all the modalities. Nonetheless, these findings suggest that some predictive factors run through different screen modalities while others are associated with a specific screen modality. A previous study reported that boys reported higher overall screen time primarily attributed to video games and videos while girls reported more time texting, social networking, and video chatting (Nagata et al., 2022).

## 5 Limitations and recommendations

Despite the strengths of this study, including the use of repeated cross-sectional data over six years with large, representative samples (n > 2300 per wave), several limitations warrant consideration. First, the cross-sectional design precludes causal inference; observed associations should be interpreted as correlational. Future longitudinal or experimental studies are needed to clarify temporal and potentially bidirectional relationships between screen time, behavioral factors, and mental well-being. Second, the sample was limited to upper secondary school students (primarily aged 16–17), which restricts the generalizability of findings to younger adolescents and children. Future research should include a broader age range to examine developmental differences in screen time behaviors and their correlates. Third, although validated and widely used instruments were employed, some measures relied on single items or self-reports, which may have introduced reporting bias. Incorporating objective assessments (e.g., digital tracking, actigraphy) and multi-informant reports could strengthen future studies. Fourth, as screen-use domains may occur simultaneously, summing midpoint estimates across modalities may overestimate total screen exposure and does not capture multitasking. Accordingly, the composite should be interpreted as an aggregated exposure indicator rather than precise time use. Fifth, the findings should be interpreted in the context of a region (i.e., Jönköping County), school-based sample from one Swedish county and may not fully generalize to all adolescents nationally or internationally. Sixth, the study was not preregistered, and the analyses should be interpreted as observational and primarily exploratory. Although we specified a consistent covariate set and used pooled models across survey waves, we evaluated multiple predictors and interactions; therefore, p-values should be interpreted as descriptive measures of the evidence rather than strictly confirmatory tests. Future work could preregister analytic plans and replication hypotheses. Finally, the study focused on weekday screen time after school hours and did not differentiate between content quality or context of use (e.g., educational vs. entertainment). Moreover, weekend screen use was not assessed; therefore, results may not reflect total weekly screen exposure. Future research should consider these qualitative aspects to better understand which types of screen engagement may be more detrimental or protective for adolescents’ well-being.

## 6 Conclusion

The present study examined the trends and correlates of excessive screen time among Swedish adolescents across 2017, 2020 and 2023. Screen time remained high across survey waves, with a consistently high prevalence of excessive screen use (86–88%). Victimization (e.g., being bullied or harassed) and psychosomatic symptoms decreased over time. Sleep duration, snus use, and regular physical activity varied across survey waves. In general, school absenteeism, alcohol use, smoking, psychosomatic symptoms, shorter sleep duration, being male, not living with both parents, and less physical activity were associated with higher screen time. However, only sleep duration, living with both parents, physical activity, and school absenteeism were consistently associated with all modalities (i.e., film/TV, gaming, and social media). These suggest that there are several factors of screen time among Swedish adolescents which cuts across sociodemographic, psychosocial, behavioral and health-related factors. Therefore, parents, health professionals and researchers need to pay more attention to screen use and take into consideration the lived experiences of adolescents. Nonetheless, to help address potential consequences of extended screen time, health professionals and educators must continue to inform the populace and especially older adolescents of extended use and dependence on digital devices for daily activities.

## CRediT authorship contribution statement

**Amir Pakpour:** Writing – review & editing, Writing – original draft, Visualization, Validation, Methodology, Formal analysis, Data curation, Conceptualization. **Karina Huus:** Writing – review & editing, Validation, Supervision, Methodology. **Daniel Kwasi Ahorsu:** . **Gunilla Björling:** Writing – review & editing, Supervision, Methodology, Conceptualization. **Anders Broström:** Writing – review & editing, Validation, Supervision, Methodology. **Staffan Bengtsson:** Writing – review & editing, Validation, Supervision, Methodology. **Malin Jakobsson:** Writing – review & editing, Validation, Supervision, Methodology, Conceptualization. **Marit Eriksson:** Writing – review & editing, Visualization, Validation, Supervision, Project administration, Methodology, Investigation, Data curation, Conceptualization.

## Declaration of competing interest

The authors declare that they have no known competing financial interests or personal relationships that could have appeared to influence the work reported in this paper.

## Data Availability

The authors do not have permission to share data.
