# Supplementary material for: Trends and Behavioral Correlates of Excessive Screen Time Among Swedish Adolescents: A Repeated Cross-Sectional Study (2017–2023)
Source: Addict Behav Rep. 2026 Jan 29;23:100672. doi: 10.1016/j.abrep.2026.100672 (PMC12914245; doi:10.1016/j.abrep.2026.100672)
Supplement: Supplementary Data 1 [file mmc1.docx]

Table S1a. Pooled GLM predicting Film/TV viewing (weekday after-school, hours)

| **Predictor** | **B** | **SE** | **95% CI** | **p-value** |
| --- | --- | --- | --- | --- |
| **Survey year (ref: 2017)** |  |  |  |  |
| 2023 vs 2017 | −0.239 | 0.320 | −0.866 to 0.387 | .454 |
| 2020 vs 2017 | −0.052 | 0.329 | −0.697 to 0.593 | .875 |
| **Substance use** |  |  |  |  |
| Narcotics use | 0.235 | 0.089 | 0.061 to 0.410 | .008 |
| Alcohol use | 0.066 | 0.078 | −0.087 to 0.219 | .400 |
| Smoking | 0.096 | 0.060 | −0.022 to 0.213 | .111 |
| Snus use | −0.018 | 0.053 | −0.122 to 0.087 | .743 |
| **Sleep & health** |  |  |  |  |
| Sleep duration (hours) | −0.108 | 0.031 | −0.169 to −0.047 | <.001 |
| Psychosomatic symptoms | 0.020 | 0.004 | 0.013 to 0.027 | <.001 |
| **Sociodemographic & psychosocial** |  |  |  |  |
| Gender | 0.027 | 0.077 | −0.124 to 0.177 | .728 |
| Living with both parents | −0.141 | 0.045 | −0.229 to −0.053 | .002 |
| Psychosocial trust | −0.014 | 0.015 | −0.043 to 0.015 | .353 |
| School absenteeism | 0.212 | 0.044 | 0.125 to 0.299 | <.001 |
| Victimization | 0.011 | 0.083 | −0.152 to 0.175 | .891 |
| Violence exposure | −0.014 | 0.095 | −0.200 to 0.172 | .880 |
| Physical activity | −0.333 | 0.043 | −0.416 to −0.249 | <.001 |
| **Interactions (omnibus tests)** | — | — | — |  |
| Year × Gender | — | — | — | .749 |
| Year × Alcohol use | — | — | — | .142 |
| Year × Sleep duration | — | — | — | .890 |

Model fit: R² = 0.065 (Adjusted R² = 0.062)

Means (hours): 2017 = 2.59; 2020 = 2.48; 2023 = 2.31

Table S1b. Pooled GLM predicting Gaming (weekday after school, hours)

| **Predictor** | **B** | **SE** | **95% CI** | **p-value** |
| --- | --- | --- | --- | --- |
| **Survey year (ref: 2017)** |  |  |  |  |
| 2023 vs 2017 | −0.580 | 0.322 | −1.212 to 0.052 | .072 |
| 2020 vs 2017 | −0.121 | 0.332 | −0.771 to 0.530 | .716 |
| **Substance use** |  |  |  |  |
| Narcotics use | 0.150 | 0.089 | −0.025 to 0.325 | .093 |
| Alcohol use | −0.174 | 0.079 | −0.329 to −0.019 | .028 |
| Smoking | 0.015 | 0.060 | −0.104 to 0.133 | .808 |
| Snus use | −0.016 | 0.054 | −0.121 to 0.089 | .763 |
| **Sleep & health** |  |  |  |  |
| Sleep duration (hours) | −0.135 | 0.031 | −0.196 to −0.073 | <.001 |
| Psychosomatic symptoms | 0.000 | 0.004 | −0.007 to 0.007 | .913 |
| **Sociodemographic & psychosocial** |  |  |  |  |
| Gender | −1.357 | 0.077 | −1.509 to −1.205 | <.001 |
| Living with both parents | −0.204 | 0.045 | −0.292 to −0.115 | <.001 |
| Psychosocial trust | −0.026 | 0.015 | −0.056 to 0.003 | .082 |
| School absenteeism | 0.124 | 0.044 | 0.037 to 0.211 | .005 |
| Victimization | 0.168 | 0.084 | 0.004 to 0.332 | .045 |
| Violence exposure | 0.205 | 0.096 | 0.018 to 0.393 | .032 |
| Physical activity | −0.475 | 0.043 | −0.559 to −0.391 | <.001 |
| **Interactions (omnibus tests)** | — | — | — |  |
| Year × Gender | — | — | — | .004 |
| Year × Alcohol use | — | — | — | .986 |
| Year × Sleep duration | — | — | — | .623 |

Model fit: R² = 0.176 (Adjusted R² = 0.173)

Means (hours): 2017 = 1.80; 2020 = 1.73; 2023 = 1.61

Table S1c. Pooled GLM predicting social media use (weekday after-school, hours)

| **Predictor** | **B** | **SE** | **95% CI** | **p-value** |
| --- | --- | --- | --- | --- |
| **Survey year (ref: 2017)** |  |  |  |  |
| 2023 vs 2017 | −0.397 | 0.390 | −1.162 to 0.368 | .309 |
| 2020 vs 2017 | −0.386 | 0.402 | −1.174 to 0.401 | .336 |
| **Substance use** |  |  |  |  |
| Narcotics use | −0.145 | 0.109 | −0.358 to 0.068 | .182 |
| Alcohol use | 0.622 | 0.096 | 0.435 to 0.810 | <.001 |
| Smoking | 0.610 | 0.073 | 0.466 to 0.753 | <.001 |
| Snus use | 0.259 | 0.065 | 0.131 to 0.386 | <.001 |
| **Sleep & health** |  |  |  |  |
| Sleep duration (hours) | −0.168 | 0.038 | −0.242 to −0.094 | <.001 |
| Psychosomatic symptoms | 0.022 | 0.004 | 0.013 to 0.030 | <.001 |
| **Sociodemographic & psychosocial** |  |  |  |  |
| Gender | 0.602 | 0.094 | 0.419 to 0.786 | <.001 |
| Living with both parents | −0.139 | 0.055 | −0.246 to −0.032 | .011 |
| Psychosocial trust | −0.013 | 0.018 | −0.049 to 0.023 | .471 |
| School absenteeism | 0.240 | 0.054 | 0.134 to 0.346 | <.001 |
| Victimization | 0.005 | 0.102 | −0.194 to 0.205 | .958 |
| Violence exposure | −0.198 | 0.116 | −0.424 to 0.029 | .087 |
| Physical activity | −0.240 | 0.052 | −0.342 to −0.138 | <.001 |
| **Interactions (omnibus tests)** | — | — | — |  |
| Year × Gender | — | — | — | .003 |
| Year × Alcohol use | — | — | — | .005 |
| Year × Sleep duration | — | — | — | .140 |

Model fit: R² = 0.122 (Adjusted R² = 0.119)

Means (hours): 2017 = 3.18; 2020 = 2.97; 2023 = 3.00


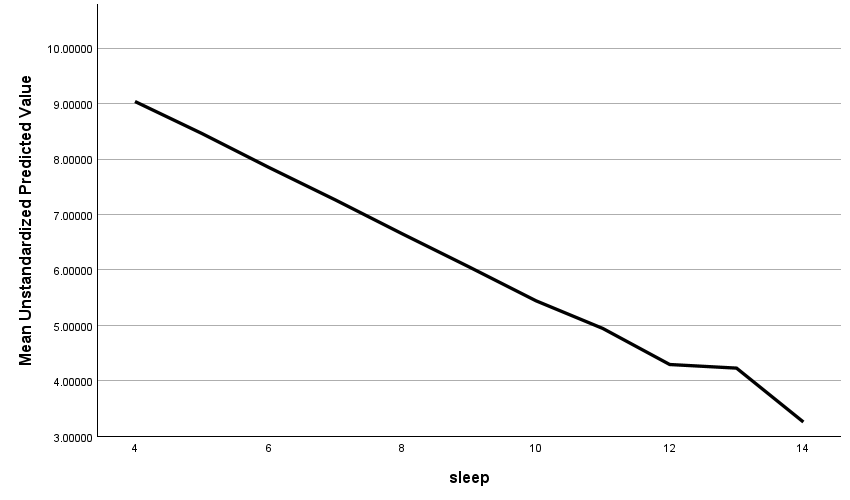


Supplementary Figure S1. Mean predicted weekday after-school screen time (hours) across levels of sleep duration, derived from the multivariable linear regression model and adjusted for alcohol use, gender, survey year, and other covariates.

**Supplementary Table S2A. Sensitivity analysis using multiple imputation (MICE): pooled regression estimates compared with complete-case analysis**

*Outcome: total screen time (hours). MI: pooled estimates from 20 imputations; CC: complete-case model.*

| **Predictor** | **MI B (95% CI)** | **MI p** | **CC B** | **CC p** |
| --- | --- | --- | --- | --- |
| 2020 vs 2017 | -0.916 [-2.322, 0.491] | 0.201 | -0.519 | 0.502 |
| 2023 vs 2017 | -1.554 [-2.897, -0.212] | 0.023 | -1.155 | 0.124 |
| Alcohol use | 0.768 [0.453, 1.083] | <0.001 | 0.578 | 0.002 |
| Smoking | 0.625 [0.380, 0.869] | <0.001 | 0.758 | <0.001 |
| Snus use | 0.172 [-0.052, 0.397] | 0.132 | 0.209 | 0.095 |
| Narcotics use | 0.372 [0.020, 0.724] | 0.038 | 0.235 | 0.258 |
| Sleep duration (hours) | -0.421 [-0.551, -0.291] | <0.001 | -0.405 | <0.001 |
| Psychosomatic symptoms | 0.044 [0.027, 0.060] | <0.001 | 0.040 | <0.001 |
| Female gender | -0.887 [-1.207, -0.567] | <0.001 | -0.723 | <0.001 |
| Living with both parents | -0.497 [-0.695, -0.300] | <0.001 | -0.487 | <0.001 |
| School absenteeism | 0.560 [0.380, 0.740] | <0.001 | 0.574 | <0.001 |
| Physical activity | -1.040 [-1.225, -0.855] | <0.001 | -1.054 | <0.001 |
| Psychosocial trust | -0.052 [-0.113, 0.008] | 0.088 | -0.056 | 0.110 |
| Victimization | 0.157 [-0.173, 0.487] | 0.351 | 0.197 | 0.310 |
| Exposure to violence | 0.235 [-0.111, 0.581] | 0.182 | 0.030 | 0.891 |
| 2020 × Female gender | -0.033 [-0.425, 0.360] | 0.871 | -0.208 | 0.372 |
| 2023 × Female gender | 0.524 [0.124, 0.925] | 0.010 | 0.394 | 0.078 |
| 2020 × Alcohol use | -0.305 [-0.717, 0.107] | 0.147 | -0.145 | 0.550 |
| 2023 × Alcohol use | -0.733 [-1.145, -0.321] | <0.001 | -0.551 | 0.018 |
| 2020 × Sleep duration | 0.093 [-0.090, 0.275] | 0.319 | 0.035 | 0.720 |
| 2023 × Sleep duration | 0.138 [-0.033, 0.309] | 0.113 | 0.112 | 0.241 |

**Notes.** MI = multiple imputation by chained equations (20 imputations, 20 iterations). Continuous variables were imputed using predictive mean matching and binary variables using logistic regression. Estimates were pooled using Rubin’s rules. CC = complete-case analysis.

**Supplementary Table S3A. Sensitivity analysis (binary outcome): High screen time (>3 hours/day)**

| **Predictor** | **CC OR (95% CI)** | **CC p** | **MI OR (95% CI)** | **MI p** |
| --- | --- | --- | --- | --- |
| 2020 vs 2017 (year2) | 1.09 [0.60, 1.99] | 0.766 | 1.01 [0.62, 1.63] | 0.967 |
| 2023 vs 2017 (year3) | 0.92 [0.47, 1.81] | 0.805 | 0.75 [0.45, 1.23] | 0.245 |
| Alcohol use (yes) | 2.19 [1.72, 2.78] | <0.001 | 2.13 [1.73, 2.63] | <0.001 |
| Smoking (yes) | 1.53 [0.97, 2.42] | 0.070 | 1.17 [0.83, 1.65] | 0.363 |
| Snus use (yes) | 0.98 [0.69, 1.38] | 0.889 | 0.87 [0.65, 1.17] | 0.360 |
| Narcotics use (yes) | 0.53 [0.31, 0.93] | 0.027 | 0.79 [0.48, 1.31] | 0.370 |
| Sleep duration (hours) | 0.82 [0.74, 0.91] | <0.001 | 0.83 [0.76, 0.90] | <0.001 |
| Psychosomatic symptoms | 1.04 [1.02, 1.06] | <0.001 | 1.03 [1.02, 1.05] | <0.001 |
| Female gender | 0.82 [0.65, 1.04] | 0.108 | 0.86 [0.69, 1.06] | 0.162 |
| Living with both parents | 0.97 [0.74, 1.26] | 0.797 | 0.89 [0.70, 1.13] | 0.342 |
| School absenteeism | 1.45 [1.09, 1.94] | 0.011 | 1.48 [1.16, 1.88] | 0.002 |
| Physical activity | 0.64 [0.50, 0.84] | 0.001 | 0.75 [0.59, 0.95] | 0.015 |
| Psychosocial trust | 1.00 [0.91, 1.10] | 0.987 | 1.03 [0.96, 1.11] | 0.426 |
| Victimization | 1.21 [0.70, 2.11] | 0.495 | 1.37 [0.87, 2.15] | 0.180 |
| Exposure to violence | 1.26 [0.66, 2.42] | 0.486 | 1.49 [0.94, 2.35] | 0.090 |

CC = complete-case logistic regression.

MI = pooled logistic regression across 20 imputed datasets (MICE), combined using Rubin’s rules.

ORs are adjusted for all covariates shown.

CC confidence intervals are Wald CIs; MI CIs are from pooled model estimates.

**Supplementary Table S3B. Sensitivity analysis (binary outcome): High screen time (>5 hours/day)**

| **Predictor** | **CC OR (95% CI)** | **CC p** | **MI OR (95% CI)** | **MI p** |
| --- | --- | --- | --- | --- |
| 2020 vs 2017 (year2) | 0.92 [0.67, 1.27] | 0.624 | 0.97 [0.75, 1.25] | 0.811 |
| 2023 vs 2017 (year3) | 1.05 [0.74, 1.50] | 0.776 | 0.95 [0.73, 1.24] | 0.709 |
| Alcohol use (yes) | 1.34 [1.17, 1.54] | <0.001 | 1.38 [1.23, 1.55] | <0.001 |
| Smoking (yes) | 1.47 [1.17, 1.83] | <0.001 | 1.29 [1.07, 1.54] | 0.006 |
| Snus use (yes) | 1.19 [0.99, 1.42] | 0.067 | 1.14 [0.97, 1.34] | 0.104 |
| Narcotics use (yes) | 1.06 [0.76, 1.49] | 0.738 | 1.09 [0.82, 1.44] | 0.562 |
| Sleep duration (hours) | 0.81 [0.77, 0.86] | <0.001 | 0.84 [0.80, 0.88] | <0.001 |
| Psychosomatic symptoms | 1.02 [1.01, 1.03] | 0.002 | 1.02 [1.01, 1.03] | 0.002 |
| Female gender | 0.94 [0.82, 1.08] | 0.381 | 0.92 [0.82, 1.04] | 0.195 |
| Living with both parents | 0.73 [0.62, 0.85] | <0.001 | 0.74 [0.65, 0.85] | <0.001 |
| School absenteeism | 1.29 [1.11, 1.50] | <0.001 | 1.31 [1.16, 1.49] | <0.001 |
| Physical activity | 0.59 [0.51, 0.68] | <0.001 | 0.62 [0.55, 0.70] | <0.001 |
| Psychosocial trust | 1.03 [0.98, 1.09] | 0.213 | 1.03 [0.99, 1.08] | 0.127 |
| Victimization | 1.30 [0.97, 1.73] | 0.080 | 1.21 [0.96, 1.52] | 0.106 |
| Exposure to violence | 1.07 [0.77, 1.49] | 0.693 | 1.14 [0.89, 1.47] | 0.283 |

CC = complete-case logistic regression.

MI = pooled logistic regression across 20 imputed datasets (MICE), combined using Rubin’s rules.

ORs are adjusted for all covariates shown.

CC confidence intervals are Wald CIs; MI CIs are from pooled model estimates.
